# Oligo-Carrageenan Kappa-Induced Reducing Redox Status and Increase in TRR/TRX Activities Promote Activation and Reprogramming of Terpenoid Metabolism in *Eucalyptus* Trees

**DOI:** 10.3390/molecules19067356

**Published:** 2014-06-05

**Authors:** Alberto González, Marlen Gutiérrez-Cutiño, Alejandra Moenne

**Affiliations:** 1 Marine Biotechnology Laboratory, Faculty of Chemistry and Biology, University of Santiago of Chile, Santiago 9170022, Chile; E-Mail: alberto.ngf@gmail.com; 2 Molecular Magnetism Laboratory, Faculty of Chemistry and Biology, University of Santiago of Chile, Santiago 9170022, Chile; E-Mail: marlen.gutierrez@usach.cl

**Keywords:** ascorbate, *Eucalyptus globulus*, glutathione, NADPH, oligo-carrageenan kappa, redox status, secondary metabolism, terpenoids, thioredoxin

## Abstract

In order to analyze whether the reducing redox status and activation of thioredoxin reductase (TRR)/thioredoxin(TRX) system induced by oligo-carrageenan (OC) kappa in *Eucalyptus globulus* activate secondary metabolism increasing terpenoid synthesis, trees were sprayed on the leaves with water, with OC kappa, or with inhibitors of NAD(P)H, ascorbate (ASC) and (GSH) synthesis and TRR activity, CHS-828, lycorine, buthionine sulfoximine (BSO) and auranofine, respectively, and with OC kappa and cultivated for four months. The main terpenoids in control *Eucalyptus* trees were eucalyptol (76%), α-pinene (7.4%), aromadendrene (3.6%), silvestrene (2.8%), sabinene (2%) and α-terpineol (0.9%). Treated trees showed a 22% increase in total essential oils as well as a decrease in eucalyptol (65%) and sabinene (0.8%) and an increase in aromadendrene (5%), silvestrene (7.8%) and other ten terpenoids. In addition, treated *Eucalyptus* showed seven *de novo* synthesized terpenoids corresponding to carene, α-terpinene, α-fenchene, γ-maaliene, spathulenol and α-camphenolic aldehyde. Most increased and *de novo* synthesized terpenoids have potential insecticidal and antimicrobial activities. Trees treated with CHS-828, lycorine, BSO and auranofine and with OC kappa showed an inhibition of increased and *de novo* synthesized terpenoids. Thus, OC kappa-induced reducing redox status and activation of TRR/TRX system enhance secondary metabolism increasing the synthesis of terpenoids and reprogramming of terpenoid metabolism in *Eucalyptus* trees.

## 1. Introduction

Terpenoids (terpenes and oxygenated terpenes) are synthesized by condensation of the five carbon precursor isopentenyl diphosphate (IPP) or its isomer dimethylallyl diphosphate (DMAPP) [1]. IPP is synthesized through the mevalonate pathway occurring in the cytosol as well as through the non-mevalonate pathway in chloroplasts [2,3]. The sequential head to tail addition of IPP and DMAPP leads to the formation of geranyl diphosphate (GPP), farnesyl diphosphate (FPP) and geranylgeranyl diphosphate (GGPP), which are the precursors of monoterpenes, sesquiterpenes and diterpenes, respectively [3]. Monoterpenes and sesquiterpenes are volatile compounds which are involved in plant-plant, plant-insect and plant-animal interactions [1,4].

**Figure 1:**
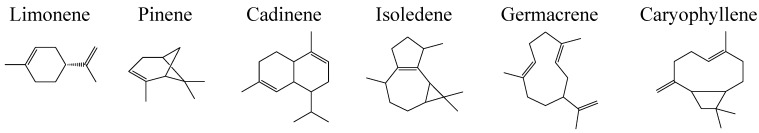
Precursors of mono- and sesquiterpenes.

The precursors GPP and FPP are the substrate of hundreds of monoterpene and sesquiterpene synthases (TS) that are differentially expressed in diverse plant species and tissues [5,6]. In general, a mono- or sesquiterpene synthase synthesizes a single mono- or sesquiterpene, but enzymes synthesizing more than one mono- or sesquiterpenes have also been described [7]. The simplest mono-, sesqui- and diterpene skeleton can be further modified by enzymes such as hydroxylases, dehydrogenases, reductases or glycosyl-, methyl- and acyl-transferases leading to the synthesis of many thousands of different terpenoids [1]. The principal families of monoterpenes are derived from limonene and pinene, and those of sesquiterpenes are derived from aromadendrene, cadinene, germacrene and caryophyllene, among others (Figure 1).

*Eucalyptus* species belong to the family Myrtaceae which originated in Australia where more than 600 species have been identified [8]. *Eucalyptus globulus* trees display rapid growth and they have been introduced in many countries with temperate weather as a source for cellulose extraction, building material and to obtain essential oils from the leaves for pharmaceutical uses. Leaves of *E. globulus* trees contain essential oils constituted by terpenoids, mainly by the monoterpenes eucalyptol (72.7%), α-pinene (9.2%), α-terpineol (2.5%) and the sesquiterpene aromadendrene (2.5%) having antioxidant, anti-inflammatory and antimicrobial properties [9,10,11]. In this sense, it has been shown that eucalyptol has antiviral, antibacterial and antifungal activities *in vitro* [12,13,14]. In addition, eucalyptol and aromadendrene have synergistic effects in regard to antioxidant and antimicrobial properties [14]. In addition, eucalyptol has larvicidal effects against larvae of the mosquito *Aedes aegipti* [15], α-pinene has a repellent effect against pine bark beetle [16] and α-terpinene has larvicidal effect against *A. aegypti* and *A. albopictus* larvae [17]. Thus, the most abundant mono- and sesquiterpenes in leaves of *Eucalyptus* trees have antimicrobial and larvicidal effects which may protect trees against microbes and insects.

Oligo-carrageenan (OC) kappa is prepared by acid hydrolysis of pure kappa carrageenan obtained from marine red algae. OC kappa is constituted by 20 units of galactose linked by alternate β-1,4- and α-1,3-glycosidic bonds with sulfate groups located in positions 2, 4 and 6 of the galactose ring with anhydrogalactose units (for a model see [18]). In a previous work, we determined that OC kappa increased the growth of *E. globulus* trees cultivated for three years in the field without additional treatment, as well as the amount of total essential oils and polyphenolic compounds [19]. Recently, we showed that OC kappa induced an increase in the level of the reducing compounds NADPH, ascorbate (ASC) and glutathione (GSH) as well as in thioredoxin reductase (TRR) and thioredoxin (TRX) activities [20]. The increase in reducing compounds changed the redox status to a more reducing condition, the increase in NADPH activate TRR/TRX activities which, in turn, activate photosynthesis, C, N and S assimilation, basal metabolism and growth in *E. globulus* trees [20].

In this work, we analyzed whether the reducing redox status induced by OC kappa in *Eucalyptus* trees activates secondary metabolism, in particular, terpenoid synthesis. To this end, we used inhibitors of NADPH, ASC and GSH synthesis and TRR activity corresponding to CHS-828, lycorine, buthionine sulfoximine and auranofine, respectively. In addition, we identified terpenoids that decreased, increased or were synthesized *de novo* in response to OC kappa having potential antimicrobial, repellent or insecticidal activities.

## 2. Results and Discussion

### 2.1. Main Terpenoids in the Leaves of Control Eucalyptus Trees

The main monoterpenoids of the limonene family in essential oils of control *Eucalyptus* leaves were eucalyptol (76%), silvestrene (2.8%), sabinene (2%) and α-terpineol (0.9%) and their levels were 3,757, 142, 101 and 45 μg·g^−1^ of fresh tissue (FT), respectively, and the monoterpeneoid of pinene family, α-pinene (7.4%), and its level was 499 μg·g^−1^ of FT. The main sesquiterpenoid of the cadinene family was δ-cadinene and its level 29 μg·g^−1^ of FT and the sesquiterpenoids of the isoledene family were aromadendrene (3.6%) and isoledene and their levels were 180 and 26 μg·g^−1^ of FT, respectively. Thus, the main terpenoids present in essential oils of control *E. globulus* trees were eucalyptol (76%), α-pinene (7.4%), aromadendrene (3.6%), silvestrene (2.8%), sabinene (2%) and α-terpineol (0.9%). The latter data is relatively in accord with results obtained in *E. globulus* cultivated in the Yunnan province of Southern China where the main terpenoids were eucalyptol (72.7%), α-pinene (9.2%), α-terpineol (3.1%) and aromadendrene (2.5%) [9]. In addition, these results slightly differ from those obtained in *E. globulus* cultivated in the north-eastern part of Morocco where the main terpenoids were eucalyptol (79.9%), *p*-cymene (5.14%), γ-terpinene (3.9%), α-pinene (0.7%) and α-terpineol (0.3%) and aromandendre, sabinene and silvestrene were not detected [21]. The latter suggests that the same *Eucalyptus* species cultivated in different soils and with different weather differ in the composition of main terpenoids constituting essential oils.

### 2.2. OC Kappa Induced an Increase in Total Essential Oils and a Reprogramming of Terpenoid Metabolism

The level of total essential oils in leaves of control *Eucalyptus* trees was 4.5 mg·g^−1^ of FT and in trees treated with OC kappa it was 5.5 mg·g^−1^ of FT, which corresponds to an increase of 22% at four months after treatment at 4 months after treatment. In addition, the level of six terpenoids decreased in *Eucalyptus* trees treated with OC kappa (Table 1). The monoterpenoids from limonene family, eucalyptol, sabinene and α-terpineol, decreased in treated *Eucalyptus* trees from 3757 to 3485, 101 to 42 and 499 to 457 µg·g^−1^ of FT, respectively, and the monoterpenoid of pinene family, α-pinene, decreased from 499 to 457 µg·g^−1^ of FT. Moreover, the level of the sesquiterpenoid of cadinene family, δ-cadinene, decreased from 29 to 14 µg·g^−1^ of FT and that of isoledene family, isoledene decreased from 26 to 11 µg·g^−1^ of FT (Table 1).

**Table 1:** Levels of the main terpenoids in leaves of control *Eucalyptus* and in trees treated with OC kappa.

Compound	Level in Control Trees (µg·g^−1^ FT)	Level in Treated Trees (µg·g^−1^ FT)	% of Decrease
Eucalyptol	3757 ± 188	3485 ± 174	7.2
Sabinene	101 ± 8.1	42 ± 3.3	59
α-Terpineol	45 ± 3.6	39 ± 3.1	13
α-Pinene	499 ± 25	457 ± 23	8
δ-Cadinene	29 ± 2.3	14 ± 1.1	52
Isoledene	26 ± 2.1	11 ± 0.9	59

Regarding the increase in total essential oils in *Eucalyptus* trees treated with OC kappa, we have previously determined that treated trees cultivated in the field for three years showed an increase in essential oils of 72% compared to controls [19]. This indicates that the effect of OC kappa persists after three years and that the level of total essential oils increased with time in treated *Eucalyptus* trees. Regarding terpenoids that decreased in treated *Eucalyptus* trees such as eucalyptol, sabinene, α-terpineol, α-pinene, δ-cadinene and isoledene they are probably the precursors that are consumed to synthesize terpenoids that increased or that were synthesized *de novo* in treated trees.

On the other hand, the level of 12 terpenoids increased in the leaves of treated *Eucalyptus* trees (Table 2). Monoterpenoids from the limonene family, silvestrene, α-phellandrene, γ-terpinene and limonene oxide, increased from 142 to 419, 9 to 27, 9 to 22 and 7 to 12 µg·g^−1^ of FT, respectively, and that of monoterpenoid of the pinene family, β-pinene, increased from 28 to 66 µg·g^−1^ of FT. The sequiterpenoid of the cadinene family, γ-cadinene, increased from 4 to 5 µg·g^−1^ of FT and those of the isoledene family, aromadendrene, viriflorene, α-gurjunene, γ-gurjunene and α-guaiene increased from 180 to 267, 39 to 82, 33 to 208, 16 to 78 and 1 to 6 µg·g^−1^ of FT, respectively. In addition, the linear monoterpene myrcene increased from 42 to 53 µg·g^−1^ of FT in treated trees (Table 2).

**Table 2:** Level of terpenoids that increased in leaves of *Eucalyptus* trees treated with OC kappa.

Compound	Level in Control Trees (µg·g^−1^ FT)	Level in Treated Trees (µg·g^−1^ FT)	% of Increase
Silvestrene	142 ± 7	419 ± 21	195
α-Phellandrene	9 ± 0.7	27 ± 2.2	193
γ-Terpinene	9 ± 0.7	22 ± 1.8	151
Limonene oxide	7 ± 0.6	12 ± 1	70
β-Pinene	28 ± 2.2	66 ± 5.3	135
γ-Cadinene	4 ± 0.3	5 ± 0.4	44
Aromadendrene	180 ± 9	267 ± 13.4	49
Viridiflorene	39 ± 3.1	82 ± 6.6	110
α-Gurjunene	33 ± 2.6	208 ± 17	529
γ-Gurjunene	16 ± 1.3	78 ± 6.3	390
α-Guaiene	1 ± 0.04	6 ± 0.5	1041
Myrcene	42 ± 3.4	53 ± 4.3	26

Regarding tepenoids that increased in response to OC kappa, it is important to mention that the monoterpene γ-terpinene showed insecticidal activity *in vitro* against the red flour beetle *Tribolium castaneaum* (Coleoptera) [22]. In addition, the monoterpene β-pinene displayed insecticidal activity against the rice weevil *Sitophilus oryzea* (Coleoptera) [23]. Moreover, the sequiterpene aromadendrene has antibacterial activity and showed a synergistic effect with eucalyptol [13,14]. Thus, the increase in terpenoids induced by OC kappa may enhance defense against insects and microbes in *Eucalyptus* trees.

In addition, six terpenoids were synthesized *de novo* the leaves of treated *Eucalyptus* trees. The monoterpenoids of the limonene family, carene and α-terpinene, and that of pinene family, fenchene were synthesized *de novo* in treated trees. The sequiterpenoid of cadinene family, γ-maaliene, that of isoledene family, spathulenol, as well as the monoterpenoid related to camphenol, α-camphenolic aldehyde, were also synthesized *de novo* in treated *Eucalyptus* trees. The newly synthesized terpenoids reached levels ranging from 3 to 9 µg·g^−1^ of FT (Table 3).

**Table 3:** Level of *de novo* synthesized terpenoids in leaves of *Eucalyptus* trees treated with OC kappa.

Compound	Type	Level in Treated Trees (µg·g^−1^ FT)
Carene	Monoterpene	4 ± 0.3
α-Terpinene	Monoterpene	3 ± 0.3
α-Fenchene	Monoterpene	8 ± 0.6
γ-Maaliene	Sesquiterpene	9 ± 0.8
Spathulenol	Sesquiterpene	5 ± 0.4
α-Camphenolic aldehyde	Monoterpene	6 ± 0.4

Regarding terpenoids synthesized *de novo* in response to OC kappa, it has been shown that the monoterpene carene has an insecticidal activity against the maize weevil *Sitophilus zeamaise* (Coleoptera) [24]. In addition, the sesquiterpenoid spathulenol had a repellent activity against the yellow fever mosquitos *Aedes aegypti* and *Anopheles stephensi*[25]. Thus, terpenoids that were synthesized *de novo* in response to OC kappa have repellent and insecticidal activities *in vitro* suggesting that treated trees may have enhanced defenses against insects. Thus, treatment with OC kappa induced an increase and reprogramming of terpenoid metabolism in *E. globulus* trees since the level of six terpenoids decreased, the level of 12 terpenoids increased and six terpenoids were synthesized *de novo* in response to OC kappa.

### 2.3. OC-Kappa Induced Reducing Redox Status Change the Level of Total Essential Oils and Induced Terpenoid Synthesis Reprogramming

**Figure 2:**
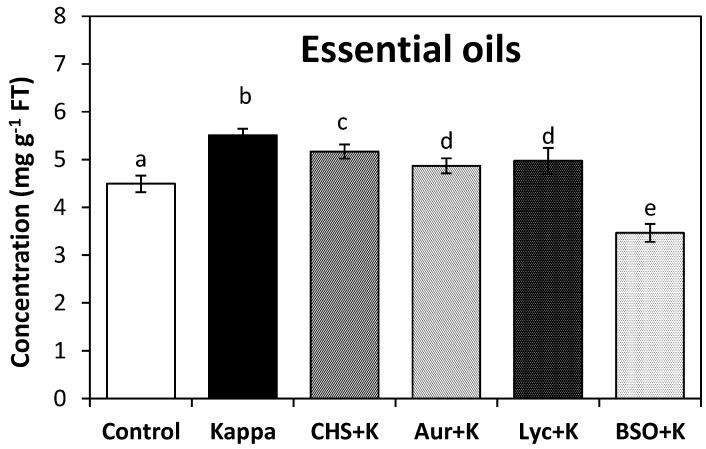
Level of total essential oils in leaves of *Eucalyptus* trees treated with water (control), with OC kappa at 1 mg·mL^−1^, or with CHS-828 and OC kappa (CHS+K), auranofine and OC kappa (Aur+K), lycorine and OC kappa (Lyc+K) and buthionine sulfoximine and OC kappa (BSO+K) and cultivated for 4 months. The level of essential oils is expressed in milligrams per gram of fresh tissue (FT). Bars represent mean values of three independent experiments and letters significant differences (*p* < 0.05).

The level of total essential oils decrease in trees treated with CHS-828, an inhibitor of NAD(P)H synthesis, auranofine, an inhibitor of TRR activity, lycorine an inhibitor of ASC synthesis, and BSO, an inhibitor of GSH synthesis, and with OC kappa from 5.5 mg·g^−1^ of FT to 5.2, 4.9, 5 and 3.5 mg·g^−1^ of FT which correspond to decreases of 34%, 63%, 53% and 100%, respectively (Figure 2). Thus, the increase in NADPH, ASC and GSH levels and in TRR/TRX activities induced by OC kappa determine, at least in part, the increase in total essential oils in *Eucalyptus* trees.

**Figure 3:**
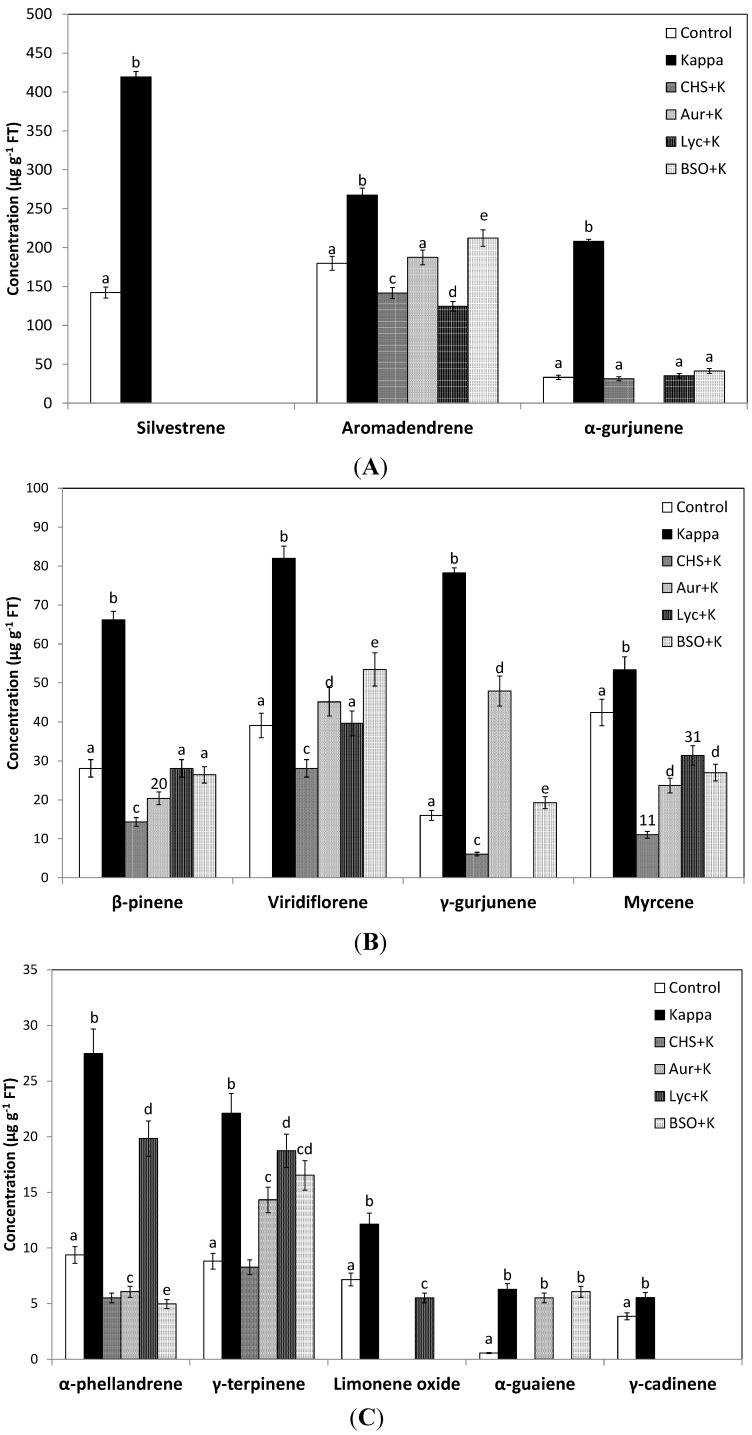
Level of terpenoids (**A**–**C**) that increased in leaves of *Eucalyptus* trees treated in control condition (control), treated with OC kappa at 1 mg·mL^−1^ (Kappa) or tretade with CHS-828 and OC kappa (CHS+K), auranofine and OC kappa (Aur+K), lycorine and OC kappa (Lyc+K) and buthionine sulfoximine and OC kappa (BSO+K) and cultivated for 4 months. Bars represent mean values of three independent experiments and letters significant differences (*p* < 0.05).

In addition, the inhibitors CHS-828, auranofine, lycorine and BSO completely inhibited the increase in the monterpenes silvestrene (Figure 3A), β-pinene (Figure 3B) and limonene oxide (Figure 3C) and that of the sequiterpene aromadrendene, α-gurjunene (Figure 3A), viridflorene, myrcene (Figure 3B) and γ-cadinene (Figure 3C). In contrast, the increase in the monoterpene γ-terpinene (Figure 3C) and the sequiterpene α-guaiene (Figure 3C) was only partially inhibited by auranofine, lycorine and BSO and the increase in γ-gurjunene (Figure 3B) was partially inhibited by auranofine and BSO. Moreover, CHS-828, auranofine, lycorine and BSO completely inhibited the increase of *de novo* synthesized terpenoids (data not shown). Thus, the increase in the synthesis of NADPH, ASC and GSH and TRR/TRX activities determine, at least in part, the increase in terpenoid synthesis and the reprogramming of terpenoid metabolism.

Regarding NADPH and terpenoid synthesis, it has been shown that the enzyme of the mevalonate pathway 3-hydro-3methylglutarate-coA (HMG-coA) reductase, which is the limiting step of the pathway, require NADPH to synthesize mevalonate. In addition, the enzyme of the non-mevalonate pathway 1-deoxy-D-xylulose 5-P (DXP) reducto-isomerase requires NADPH to synthesize 2-methyl-3-erythritol 4-phosphate (MEP) [2,3]. Thus, it is not surprising that a decrease in NADPH synthesis inhibits terpenoid synthesis. Regarding TRR/TRX activities and terpenoid synthesis, it has been shown that chloroplasts TRXs *f* and *m* bind to DXP reducto-isomerase and GcpE enzyme belonging to the non-mevalonate pathway [26]. Considering that there is a cross-talk among NADPH, ASC and GSH that command the increase in TRR/TRX activities [20] and that TRX may activate at least the non-mevalonate pathway, it is possible to conclude that the increase in ASC and GSH may also regulate the increase in terpenoid synthesis. In addition, it is interesting to mention that *Eucalyptus* trees treated with OC kappa cultivated in the field for three years showed an increase in polyphenolic compounds with potential antipathogenic activities [19]. Thus, OC kappa activates the synthesis of volatile terpenoids and polyphenolic compounds with antipathogenic activities which may enhance defense against insects and microbes in *Eucalyptus* trees.

## 3. Experimental

### 3.1. Preparation of OC Kappa

Twenty grams of pure (*i.e*., free of proteins and secondary metabolites) commercial kappa2 carrageenan (Gelymar S.A., Santiago, Chile) were solubilized in water (2 L) at 60 °C. Concentrated HCl (36.2 N) was added to reach a final concentration of 0.1 N, the solution was incubated for 45 min at 60 °C and then NaOH 1 M was added to obtain pH 7. A sample of 10 µL depolymerized carrageenan (oligo-carrageenans, OC) kappa was analyzed by electrophoresis in an agarose gel (1.5% *w*/*v*) using 100 V for 1 h and dextran sulphate of 8 and 10 kDa as standards (Sigma, St Louis, MO, USA). The gel was stained with 15% *w*/*v* Alcian blue dye in 30% *v*/*v* acetic acid/water for 1 h at room temperature and washed with 50% *v*/*v* acetic acid/water for 1 h. OC kappa was visualized as a relative discrete band of around 10 kDa.

### 3.2. Treatment of Trees with OC Kappa and Inhibitors/OC Kappa

*E. globulus* trees with an initial height of 30 cm (*n* = 10 for each group) were cultivated outdoors in plastic bags containing compost. *E. globulus* trees were sprayed in the upper and lower part of the leaves with 5 mL per plant with water/methanol 9:1 *v*/*v* (control group, *n* = 10), an aqueous solution of OC kappa at a concentration of 1 mg·mL^−1^ (treated group 1, *n* = 10), a water/methanol solution of 250 μM CHS-828, an inhibitor of nicotinamide phosphoribosyltransferase [27] and NAD(P)H synthesis (treated group 2, *n* = 10), a water/methanol solution of 250 μM lycorine, an inhibitor of galactonolactone dehydrogenase [28] and of ASC synthesis (treated group 3, *n* = 10), a water/methanol solution of 1.5 mM buthionine sulfoximine (BSO), an inhibitor of γ-glutamylcysteine synthase [29] and of GSH synthesis (treated group 4, *n* = 10), and with auranofine [30], an inhibitor of TRR activity (treated group 5), and with OC kappa at a concentration of 1 mg·mL^−1^. Trees of treated groups 2, 3, 4 and 5 were initially treated twice with CHS-828, auranofine, lycorine or BSO, and after two weeks they were treated with OC kappa once a week, four times in total, cultivated without any additional treatment for 4 months. Leaves were obtained from the middle part of control and treated trees and pooled into three groups to perform further analysis (*n* = 3). The height of trees was determined using a measuring tape.

It is important to mention that the selected concentration of OC kappa (1 mg·mL^−1^) is the optimal concentration to stimulate growth in *Eucalyptus* trees, since a higher concentration of 5 mg·mL^−1^ did not further increase growth and a concentration of 10 mg·mL^−1^ inhibited the increase in growth (unpublished data). In addition, the chosen period of culture was 4 months because from three months forward the effect of OC kappa on basal metabolism was clearly evident [20]. Moreover, it is important to point out that the optimal concentration CHS-828 decreased NADPH content, lycorine inhibited galatonolactone dehydrogenase (GLDH) activity, BSO inhibited γ-glutamylcysteine synthase (γ-GCS) activity and auranofine inhibited TRR activity at four months of culture without additional treatment [20].

### 3.3. Distillation of Total Essential Oils

Leaves (50 g of fresh tissue) of control and treated *Eucalyptus* trees (*n=* 3 for each group) were homogenized in a food mill and added to distilled water (200 mL). Essential oils were distilled using a Clevenger apparatus for 30 min. Essential oils which have a lower density than water were recovered with a micropipette and weighted using a precision balance.

### 3.4. Analysis of Terpenoids by GC-MS

Analysis of terpenoids was performed using gas chromatography (GC) coupled to mass spectrometry (MS) as described by Ait-Ouazzou *et al.* [21], with some modifications. A sample of distilled essential oils (1 μL) was diluted 100 times in *n*-hexane and analyzed using a GC-MS apparatus (model Clarus 500, Perkin Elmer, Waltham, MA, USA) having silica capillary column Equity-5 of 30 m length, 0.25 mm inner diameter, 0.25 μm particle size (Supelco, Belleponte, PA, USA) at temperatures of 60 °C for 4 min, 64 °C for 2 min, 155 °C for 5 min and 250 °C for 10 min, using helium as carrier gas and a flow rate of 1 mL·min^−1^. Temperature of the injector was 50 °C and that of MS transfer line 250 °C. MS analysis was performed using an electron impact ionization of 70 eV in the *m*/*z* range of 40–400. The analysis was performed in triplicate for each sample. Identification of terpenoids was made by matching recorded mass spectra with reference spectra compiled in the computer library NIST2006 Mass Spectra Library.

### 3.5. Statistical Analysis

Significant differences were determined by two-way analysis of variance (ANOVA) followed by Tukey’s multiple comparison tests (*T*). Mean values were determined using three independent samples. Differences between mean values were considered to be significant at a probability of 5% (*p* < 0.05) [31].

## 4. Conclusions

In this work, we showed that the reducing redox status due to the increase in NADPH, ASC and GSH synthesis and the increase TRR/TRX activities induced by OC kappa in *E. globulus* trees determine the activation of secondary metabolism, leading to an increase in the synthesis of terpenoids having antimicrobial, repellent and insecticidal activities and suggesting that treated trees may have an enhanced defense against insects and microbes.
